# Purple corn cob (*Zea mays* L.) powder at different percentages on performance, fatty acid, nutrients profile, and lipoperoxidation in eggs from laying hens

**DOI:** 10.5455/javar.2025.l939

**Published:** 2025-06-02

**Authors:** Milagros Nikole Flores Nizama, Connie Gallardo Vela, Oscar Reategui Arevalo, Jimny Yoel Nuñez Delgado, Virginia Rivadeneira

**Affiliations:** 1 Faculty of Veterinary and Biological Sciences, Research Group on Management of Sustainable Animal Production Systems, Universidad Cientifica del Sur, Lima, Peru; 2 Faculty of Veterinary Medicine, Universidad Nacional Mayor de San Marcos, Lima, Peru

**Keywords:** Anthocyanins, egg quality, laying hens, lipoperoxidation, purple corn, production

## Abstract

**Objective:**

The study aimed to evaluate the effect of purple corn cob powder (PCCP) at different percentages on performance, egg quality, egg weight loss, fatty acid profile, total solids, bromatological analysis, and lipoperoxidation in eggs from laying hens.

**Materials and Methods:**

One hundred twenty-eight Hy-Line Brown hens (29–35 weeks old) were divided into four treatments (0%, 0.2%, 0.4%, and 0.6% PCCP), with eight replicates and four hens per replicate.

**Results:**

Treatments with 0.4% and 0.6% PCCP significantly increased (*p* < 0.05) feed conversion ratio, laying percentage, and egg mass weight. Similar improvements were observed for yolk weight and Haugh units. There were no differences (*p* > 0.05) in shell weight, albumen weight, shell thickness, and yolk color. Treatments with 0.4% and 0.6% PCCP increased (*p *< 0.05) unsaturated fatty acids and decreased saturated fatty acids and thiobarbituric acid reactive substance levels. Eggs stored for 28 days showed lower weight loss (*p* < 0.05) in treatments with 0.2% and 0.4% PCCP.

**Conclusion:**

PCCP inclusion in laying hens‘ diets can enhance productive indices, egg quality (both external and internal), increased unsaturated fatty acids, and help preserve egg properties during storage in the egg yolk.

## Introduction

Poultry farming is a significant livestock activity with extensive global development, providing products such as meat and eggs, which contribute a wide array of essential elements for human nutrition, such as vitamins, proteins, and minerals [1]. Eggs are highly nutritious foods containing high protein levels and vitamins such as choline, minerals, and carotenoids, including lutein and zeaxanthin [2]. The breeding of laying hens is increasing annually. It has a lesser environmental impact compared to the production of other sources of animal protein [3].

Due to productive demand, the interest in improving both the external and internal quality of the egg to complement and enhance its nutritional benefits has been reported in several studies [4,5]. Moreover, producers also seek to increase the shelf life of chicken eggs, as it is quite limited, reaching approximately 28 days, with notable differences in the first week of storage and a reduction in albumen quality and fatty acids over time [5]. Among these differences, polyunsaturated fatty acids in the egg yolk lipids are susceptible to natural oxidation caused by metabolic processes. This is of great importance due to the negative impact on the quality and nutritional value of the egg.

It has been shown that antioxidant supplements can contribute to the appearance, oxidative stability, acceptability, and storage properties of animal-origin foods. The supplements most used in the food industry include butylhydroxyanisole, butylhydroxytoluene, propyl gallate and tert-butylhydroquinone [6]. However, consumers are currently looking for the inclusion of natural products in their daily diet. Therefore, the supplementation of natural antioxidants in the diet of hens to reduce the natural lipoperoxidation occurring in the egg through exogenous agents and increasing the concentration of intrinsic antioxidants in the body of these animals is being studied [7].

Peru has a wide diversity of climates and geographic characteristics, resulting in a great variety of agricultural products, among which purple corn (*Zea mays *L.), consumed since pre-Inca times, is of note. In 2020, the national production of purple corn was 24,600 tons [8]. Purple corn is perennial throughout the year, peaking between March and May. Its chemical composition includes salicylic acid, fats, resins, saponins, sulfur, phosphorus, and phenolic compounds [9]. Among the latter group, anthocyanins are the most important due to their significant pharmacological features, which are potentially beneficial for productive performance and animal welfare [10], and while they are present in all structures of the corn plant, they are mainly concentrated in the cob [11].

Anthocyanins are the main water-soluble pigments visible to the human eye, with colors ranging from red to purple. They counteract oxidation through a mechanism of reducing free radical activity and have anti-inflammatory, anticancer, vasoprotective, cholesterol-reducing, and antimicrobial properties, among others [1]. Regarding their use in poultry production, various products with high concentrations of anthocyanins are used to improve productive indices and egg quality, stabilize oxidative processes in egg yolk, and reduce saturated fatty acids [5]. Similar effects were found with the use of blueberry leaves [12]. On the other hand, laying hens diet with grape pomace increased the proportion of polyunsaturated fatty acids in the yolk and improved the yolk lipid oxidative stability during the storage of eggs [13]. It should be noted that the implementation of agricultural by-products, with high anthocyanin content, such as grape pomace has achieved optimal productive results in the poultry industry, as they present high levels of anthocyanins increasing egg weight and decreasing levels of malondialdehyde (MDA) in plasma and glucose in serum in laying hens [14]. The use of pomegranate peel powder decreased feed conversion and increased production in Japanese quails [15].

However, the results of including anthocyanins from purple corn cob, an important by-product in the agricultural industry, in laying hens are unknown. Therefore, this study aimed to evaluate the effect of including different percentages of purple corn cob powder (PCCP) in laying hens’ diets on productive indices, quality, and differential nutrient contribution of eggs and determine the levels of anthocyanins, flavonoids, and polyphenols in the PCCP used.

## Materials and Methods

### Ethical approval

The study involving live animals was approved by the Institutional Committee for Ethics in Research with Animals and Biodiversity of the Universidad Cientifica del Sur (093-CIEI-AB-CIENTÍFICA-2020).

### Experimental design

The study was conducted at the Experimental area for laying hens and the Chemistry and Bromatology Laboratory of the Universidad Cientifica del Sur, Lima, Peru. One hundred twenty-eight Hy-line Brown hens aged 29–35 weeks, with an initial approximate weight of 1.780 ± 0.023 kg, were randomly placed in two-tier pyramid-type laying cages with compartments measuring 60 × 52 × 40 cm, equipped with a nipple drinker, individual linear feeder (13.2 × 8.5 cm), and egg collection trays. For inclusion criteria, healthy animals with phenotypic characteristics of good laying hens, such as large and bright red crests and wattles, wide body, deep abdomen, shiny plumage, and active behavior, were selected. Birds that were lethargic, with diarrhea, prolapses, or respiratory signs were excluded. Throughout the experiment, the feed and water supply were *ad libitum*. The laying hens underwent a 3-week acclimatization stage (26–28 weeks of age), consuming the same control diet before starting the experimental diets.

### Preparation and evaluation of PCCP (Zea mays L.)

The purple corn (*Zea mays* L.) used in this study was a commercial product from the Arequipa department, Peru. The selection process involved removing grains that showed any alteration, and 8 kg of purple corn was disinfected using a standardized methodology with a 5% chlorine dioxide solution (Dioxill plus, JUANDI E.I.R.L, Peru) for approximately 10 min. The corn was then shelled to access the cob, which was sliced with a knife into approximately 0.8–1 cm thickness. The slices were placed in an oven at a temperature of 40°C for 24 h and then air-dried and weighed using a 12 kg SJ-HS Series electronic balance (Labormersa, USA) with a precision of 0.01 mg and sensitivity of ±0.015%, determining the moisture content of the sample. The slices were ground using a TE-651/2 Tecnal Rotor Mill (High Tech, USA). The chemical composition of the PCCP is presented in Table 1 and was determined by proximal analysis using 200 mg of sample with the methodology established by the Association of Official Analytical Chemists [16] and a calorimetric bomb (IKA C5000, Karnataka, India).

**Table 1.** Chemical composition of the purple corn cob powder.

Compound (m/100 mg)	Sample
Moisture, %	14.23
Total protein (N × 6.25), %	3.14
Fat, %	0.36
Crude fiber, %	21.37
Ash, %	2.36
Nitrogen-free extract, %	58.24
Total energy (Kcal/kg)	3784.5

### Anthocyanin content in PCCP

For the extraction of anthocyanins, the methodology described by [17] based on differential hydrogen potential (pH) was used. The procedure was carried out using 2.5 mg of previously ground and sifted PCCP (*Zea mays* L.), to which 200 ml of 20% ethanol was added. This was then sonicated (Branson Ultrasonic Cleaner 2510 EDTH, AC/DC input 220; Merck KGaA, Darmstadt, Germany) for 2 h. The extract obtained was filtered with the help of a vacuum pump, and Whatman No.1 filter paper was used. Finally, an aliquot of the extract was taken and diluted with potassium chloride buffer (pH 1) and sodium acetate buffer (pH 4.5). Two solutions were prepared: (1) 0.1 ml of sample and 2.49 ml of potassium chloride buffer (pH 1) and (2) 0.1 ml of sample and 2.49 ml of sodium acetate buffer (pH 4.5). Subsequently, the absorbance (Spectrophotometer Micronal^®^, model B572, Sao Paulo, Brazil) at 510 and 700 nm was measured using the following formula:

where,

MW, molecular weight of 449.2 gm/mol for cyanidin-3-glucoside; DF, dilution factor; ε510, 26,900 M^−1^cm^−1^ molar extinction coefficient for cyanidin-3-glucoside; V, volume in ml of the purple corn cob extract; W, weight of the sample in mg.

### Determination of polyphenols in purple corn cob

The methodology used was described by pharmacopeias [18,19]. The procedure was carried out using 10% Folin−Ciocalteu reagent (1 ml) along with 0.1 ml of sample, left to rest for 5 min. This was followed by mixing with 1 ml of carbonate and placing it in a water bath at 45°C for 15 min. The reading was performed on the spectrophotometer at 725 nm. Based on the absorbance values obtained (Spectrophotometer Micronal^®^, model B572, Sao Paulo, Brazil), the calibration curve was prepared, considering an optical density from 0 to 0.3 mg of gallic acid from 0 to 0.005. For data analysis, the equation *y* = 54.273*x* with an *R*^2^ = 0.9998 was obtained.

### Quantification of total flavonoids in PCCP

The methodology used was described by Stalikas [20]. A sample of 250 µl was taken to which 125 µl of water and 75 µl of 5% sodium nitrite were added and left to rest for 5 min. Then, 150 µl of 10% aluminum trichloride was added and left to rest for 6 min. Finally, 0.5 ml of 2M sodium hydroxide and 275 µl of distilled water were mixed, and the absorbance was measured at 510 nm (Spectrophotometer Micronal^®^, model B572, Sao Paulo, Brazil). A calibration curve was prepared using catechin as a reference in the concentration range of 0.5–14 µg/ml (*y* = 0.0142*x*, *R*^2^ = 0.9992). The results were expressed as catechin equivalents in µg/mg by performing the calibration curve, considering an optical density from 0 to 0.6 and µg of catechin from 0 to 40. The results of the anthocyanin, polyphenol, and flavonoid content in the PCCP were obtained following the methodology of Giusti and Wrolstad [17]; Brazilian Pharmacopeia [18]; Council of Europe [19], and Stalikas [20], and they are presented in Table 2.

### Experimental diets

The study included 128 Hy-Line Brown hens aged 29–35 weeks were included and divided into four treatments with eight replicates and four hens per replicate, receiving diets with different percentages of PCCP (0%, 0.2%, 0.4%, and 0.6%). The diets were prepared based on hard yellow corn and soybean meal, according to the nutritional requirements of the line and Rostagno et al. [21], with the PCCP directly substituted for rice dust (Table 3).

### Evaluation of performance

The initial weight of the hens was individually assessed at the beginning of the experiment, followed weekly using a 30 kg capacity electronic scale (E-accura, PA2-30, Shanghai, China) with a precision of 0.5 gm. At the end of the experiment, the final weight of the hens was recorded. The following formula was used to calculate weight gain:

**Table 2.** Antioxidant content evaluation in purple corn cob powder.

Feature	Gram/powder
Anthocyanin content	20.128 mg/mg
Total polyphenol determination	19.98 ± 0.85 mg GAE/mg
Total flavonoid quantification	16.49 ± 0.12 mg QE/mg

Values expressed as mg/mg.

**Table 3.** Experimental diets for laying hens fed with different percentages of purple corn cob powder (PCCP).

	Treatments			
Ingredients	0%	0.2%	0.4%	0.6%
Corn	52.030	52.030	52.030	52.030
Wheat bran	10.750	10.750	10.750	10.750
Rice dust	0.600	0.400	0.200	--
PCCP	--	0.200	0.400	0.600
Soybean meal, 46%	20.320	20.320	20.320	20.320
Soy oil	4.490	4.490	4.490	4.490
Calcium carbonate (fine)	4.600	4.600	4.600	4.600
Calcium carbonate (coarse)	4.500	4.500	4.500	4.500
Dicalcium phosphate	1.710	1.710	1.710	1.710
Choline chloride, 60%	0.100	0.100	0.100	0.100
Sodium bicarbonate	0.120	0.120	0.120	0.120
L-lysine	0.030	0.030	0.030	0.030
DL-methionine	0.200	0.200	0.200	0.200
NaCl	0.350	0.350	0.350	0.350
Vitamin and mineral premix	0.100	0.100	0.100	0.100
Mycotoxin binder	0.100	0.100	0.100	0.100
Nutritional chemical composition				
Dry matter (%)	89.92	89.76	89.25	89.87
Metabolizable energy (kcal/kg)	2800.04	2800.03	2800.13	2800.09
Crude protein (%)	17.88	17.92	17.95	17.89
Calcium (%)	3.89	3.90	3.90	3.90
Ether extract (%)	5.31	5.31	5.31	5.31
Available phosphorus (%)	0.60	0.60	0.60	0.60
Potassium (%)	0.70	0.70	0.70	0.70
Sodium (%)	0.20	0.20	0.20	0.20

Treatments = Treatment 1-0% PCCP, Treatment 2-0.2% PCCP; Treatment 3-0.4% PCCP, and Treatment 4-0.6%; Vitamin and mineral premix = a mix of vitamins and minerals containing Retinol (Vitamin A), 10000000 IU; cholecalciferol (Vitamin D3), 3000000 IU; DL alpha tocopherol acetate (Vitamin E), 15000 IU; menadione bisulfite (Vitamin K3), 2.5 gm; thiamine (Vitamin B1), 2 gm; riboflavin (Vitamin B2), 6 gm; cyanocobalamin (Vitamin B12), 0.0012 gm; pantothenic acid (Vitamin B5), 6 gm; folic acid (Vitamin B9), 0.5 gm; niacin (Vitamin B3), 20 gm; biotin (Vitamin B7), 0.15 gm; manganese, 60 gm; zinc, 60 gm; iron, 40 gm; copper, 6 gm; iodine, 1 gm; selenium, 0.3 gm; cobalt, 0.15 gm, Mycotoxin binder = binder for aflatoxins and fumonisins (Mycofix^®^ Secure).

Total weight gain (gm) = Final weight (gm) – Initial weight (gm)

Feed consumption was evaluated weekly. The total consumption was obtained based on weekly consumption minus the remaining feed and the daily consumption over the total days elapsed. The evaluation was carried out using the following formula:

Feed consumption (kg) = Feed provided in the week (kg) – Remaining feed (kg)

The feed conversion ratio (FCR) was calculated considering the consumption (kg) and the amount of egg produced (kg):

The mortality and morbidity percentages were evaluated daily using previously prepared records. There were no deaths or culling of birds.

The egg production percentage was evaluated daily by the collection of eggs from each cage twice a day at 1,000 and 1,500 and the weight of the eggs was performed individually using a digital scale (PCE-BSH 10000, KERN Germany) with a precision of 0.2 gm. Based on the percentage of production and egg weight, the quantity of kg of egg produced was obtained. The egg mass was calculated using the following formula:

### Evaluation of external and internal egg quality

The egg evaluation of the internal and external quality of eggs was performed weekly; one egg was randomly selected per replicate each day (Totaling 56 eggs per treatment/week). Eggs were selected and stored at room temperature. The eggs were evaluated in the Chemistry and Bromatology Laboratory of the Universidad Cientifica del Sur, being previously identified on trays for each treatment. All eggs were weighed (E-accura, PA2-30, Shanghai, China) and identified, and the morphometric index was determined based on the measurement of length and width using a 15“ digital caliper (Ubermann, China) according to the following formula:

The eggs were then broken, the content was poured onto a flat surface, and the yolk color was evaluated using a DSM YolkFan (DSM-Firmenich, Wurmisweg, Kaiseraugst, Switzerland) of 15 colors. Albumen height, yolk height, and diameter were measured using a 15“ digital caliper (Ubermann, China), and the yolk index was calculated based on these measurements using the formula:

Albumen height was used to calculate the Haugh units through the relationship between the egg weight (W) and the height of the dense white (H) using the formula:

After the evaluation, the yolk was separated from the albumen, and the yolk was weighed. The eggshells were dried at room temperature [21°C ± 0.22 and 72% ± 0.49 relative humidity (RH)] for 48 h, and the external and internal membranes were detached for the subsequent measurement of the thickness of the equatorial pole of the egg, weight, and shell index. From this, the albumen weight was determined by the difference between both structures. The shell index was calculated using the formula:

### Weight loss, lipid oxidation, total solids, and bromatological analysis

For this purpose, two eggs/replicate per day were collected for 1 week (Totaling 112 eggs per treatment). The eggs were evaluated at 35 weeks old of the laying hens. For weight loss, 28 eggs per treatment were evaluated before storing them, and the other 28 eggs were stored at room temperature (21°C ± 0.22 and 72% RH), for 4 weeks for subsequent evaluation. The methodology described by Ohkawa et al. [22] was used to determine antioxidant activity in egg yolk using thiobarbituric acid reactive substances (TBARS). The eggs were cracked, the yolk was separated into a beaker, and then mixed with 2 mg of yolk along with 10 ml of 50% glacial acetic acid in water containing 0.01% pyrogallol. The sample was homogenized using a Glas-Col 099K54 homogenizer for 15 min and then centrifuged at 1000 rpm for 60 sec.

Subsequently, 2.5 ml of the sample without the supernatant was extracted into amber glass bottles. In 13 × 100 test tubes, the following reaction system was carried out: 1 ml of 0.1M pH 7.4 phosphate buffer was added along with 0.2 ml of 28 nM deoxyribose, 0.2 ml of 20 nM ascorbate, distilled water, 0.4 ml of sample, and 0.2 ml of 20 nM copper. This was then placed in a water bath at 37°C for 30 min. After that, 1 ml of 10% trichloroacetic acid and 1 ml of 0.7% thiobarbituric acid were added and then boiled for 15 min and read on the spectrophotometer at 532 nm. The results can be expressed as IC_50_; however, some authors express them as μmol/mg or μmol/100 mg of the sample, for which the molar extinction coefficient of 1.54 × 1050 M^−1^ cm should be used.

The total solids in the egg yolks were determined in 28 eggs per treatment considering the difference between 100 and the humidity percentage. The humidity percentage was obtained through the initial weight of the yolk and the weight at the end of the lyophilizing process. The eggs were manually cracked on a flat surface and the yolk was separated from the albumen and placed in 500 ml Schott jars stored at refrigeration (6°C). They were then manually mixed, trying not to form foam. Subsequently, the samples were frozen at −40°C for 6 h. Freeze drying was carried out with a Labconco FreeZone lyophilizer (Labconco Corporation, Kansas, USA). The lyophilizing time was 24 h, and the content was placed in Ziploc bags. Finally, in the nutritional composition [16] and moisture content [Association of Official Analytical Chemists (AOAC 950.46)], total energy by calorimetric bomb, total protein (AOAC 984.13), fat (AOAC 2003.05), and ash (AOAC 942.05) were analyzed.

### Evaluation of total lipids and fatty acids in egg yolk

The extraction of total lipids in egg yolk was carried out when the hens were 35 weeks old. Twenty-eight eggs per treatment of previously lyophilized egg yolk were used. Total lipids were evaluated by acid hydrolysis following the methodology proposed by the AOAC (1990) [23]. The fatty acid profile was determined following the methodology described by Wang et al*.* [24], placing 50 mg of yolk sample in a borosilicate test tube, adding 1 ml of hexane and 3 ml of 3N HCl in methanol, placed in a water bath for an hour at 95°C. The tubes were then removed and placed on a rack to cool to room temperature. Once the tubes were cool, 8 ml of 0.88% NaCl and 3 ml of hexane were added, and homogenized by shaking for 1 min. The tubes were capped and left to rest for 4 h. The supernatant was collected in dark vials, further protected with aluminum foil, and refrigerated for subsequent analysis. To determine the fatty acids in the gas chromatograph (Shimadzu GC-R1A, Illinois, USA), a standard curve was prepared using nonadecanoic methyl ester acid (19:0) from Sigma-Aldrich (Product 5377, CAS No 1731-94-8) with a purity greater than 98%, in a concentrated solution of 5,000 mgl^−1^ in hexane.

### Statistical analysis

The data were analyzed using the STATA 18 program (StataNow^™^). The normality of the data was evaluated using the Kolmogorov−Smirnov test and homogeneity of variances by Levene test. Percent data (%) were transformed to arcsine values for analysis. Variances between treatment means were evaluated using Analysis of variance, and to compare treatments, the Tukey statistical test was used, considering a significance level of *α* = 0.05.

**Table 4.** Effects of including different percentages of purple corn cob powder (PCCP) on productive performance in laying hens.

Productive performance	Treatments^1^				SEM	*p*-value
	0%	0.2%	0.4%	0.6%		
Initial weight (gm)	1944.50	1928.00	1933.25	1940.00	68.342	0.9865
Gain weight (gm)	87.00	93.75	93.75	91.25	53.594	0.9976
Total consumption (gm)	3884.38	3882.69	3817.00	3831.75	42.162	0.0920
Production (%)	79.46^b^	78.93^b^	91.08^a^	84.82^ab^	3.719	0.0019
Eggs produced (kg)	1.438^b^	1.412^b^	1.742^a^	1.564^ab^	0.0949	0.0013
FCR (gm/gm)	2.715^ab^	2.758^a^	2.195^c^	2.453^bc^	0.143	0.0004

*N* = 32 hens per treatment; Treatments = Treatment 1-0%PCCP, Treatment 2-0.2% PCCP, Treatment 3-0.4% PCCP, and Treatment 4-0.6%; ^abc^ = different letters in each average indicate significant differences between treatments with the Tukey test (*p* < 0.05).

## Results and Discussion

### Performance

Table 4 shows the results of the performance in laying hens supplemented with PCCP. Statistically significant differences (*p* < 0.05) were observed in terms of production percentage, kg of eggs produced, and FCR. Hens supplemented with 0.4% PCCP showed a higher egg production percentage compared to the control group (−11.6%); however, there were no statistical differences (*p* > 0.05) between treatments with 0.4% and 0.6% PCCP. Similarly, regarding kg of eggs produced the diet with 0.4% PCCP obtained better results compared to the control treatments and 0.2% PCCP, with no statistical differences (*p *> 0.05) between treatments with 0.4% and 0.6% PCCP. Regarding the FCR, the treatment with 0.4% PCCP achieved better feed conversion (*p* < 0.05) compared to the diet with 0% and 0.2% PCCP. However, there were no statistical differences (*p* > 0.05) with respect to the diet with 0.6% PCCP. No statistical differences (*p* > 0.05) were found in weight gain, total, and daily feed consumption.

In this study, anthocyanins present in PCCP demonstrated the capacity to improve the productive indices of laying hens. Previous literature has described that anthocyanin can positively modify livestock and poultry productivity due to their antioxidant and anti-stress capacities, which are crucial for governing the productive index of animals. The absence of these flavonoids could lead to an increase in the production of various reactive oxygen species, reactive nitrogen, peroxides, and other free radicals [25] exceeding the antioxidant buffering capacity of the animal itself and negatively affecting their health and, therefore, production.

The antioxidant characteristics of polyphenols, such as anthocyanins, are related to their chemical structures, especially in the count and location of hydroxyl groups. Nevertheless, phenols can generally act as metal antimutagens, chelating agents, and antimicrobial and anticancer agents [26,27]. Moreover, anthocyanins can improve the use of nutrients present in food, as well as provide better control of the oxidative system and, in parallel, increase the antioxidant capacity of the body‘s own systems, such as superoxide dismutase, glutathione peroxidase, and catalase [5,25,28–30], all through the activation of the transcription factor NFE2L2 [31]. Therefore, as the defense mechanism of the birds against oxidative damage is improved, they will have more energy for egg production. It is worth adding that a study conducted by Huo et al. [32] described an improvement in the growth of atrophied oviducts, which infers that it is an additional factor in the improvement of egg laying.

Literature on the use of purple corn anthocyanins in laying hens is very limited, but the present findings could be extrapolated to those in broiler chickens. The daily feed consumption numerically increased (+5.7 gm/d) with the implementation of 80 mg/kg of purple corn anthocyanins in the diet of broiler chickens, increasing the weight by 89.48 gm at the end of the experiment [25].

Other studies conducted in hens using 5%, 10%, and 15% of pomegranate seed extract [33], 2% açaí flour [5], and 1.5% grape seed [34] demonstrated improvement in the percentage of egg production (*p* < 0.05). Furthermore, the FCR has been improved with the use of 10% and 15% pomegranate seeds [35] and 2% and 4% hibiscus calyx flour [36]. On the other hand, various authors [12,14,34–37] found no significant differences in feed consumption by implementing pomegranate peel, 10% and 15% pomegranate seed extract, blueberry leaves, 4% and 6% grape pomace, 1.5% grape seed extract, hibiscus calyx flour, and goji berry leaves (*Lycium barbarum*) at 5, 10, and 20 gm/kg of feed, respectively.

Studies conducted in quails demonstrated that egg production can be statistically increased (*p* < 0.05) by 9% with the use of 0.2, and 0.4% hibiscus calyx in feed [38] and mangosteen pericarp [39], as well as 0.5 and 1 gm/l of hibiscus calyx in drinking water [40]. Conversely, there were no significant differences in egg production using 2%, 4%, and 6% of hibiscus calyx powder [13].

Similar results have been found in the inclusion of grape seed extract at 125 and 250 ppm and 4% blackberry juice in diets of chickens and ducks, with a significant increase in body weight being observed, respectively [41,42], thereby improving the FCR.

### External and internal egg quality

Table 5 presents the results regarding the external and internal quality of eggs from hens supplemented with PCCP. Regarding the external quality of the eggs, treatment with 0.4% PCCP obtained better egg weight compared to the control treatment (−5.3 gm); however, there were no statistical differences (*p* > 0.05) compared to the treatments with 0.2% and 0.6%. In addition, the treatment with 0.4% also obtained the highest result (*p* < 0.05) regarding egg mass, showing differences compared to the control treatments (12.2 gm) and with 0.2% PCCP.

On the other hand, regarding the internal quality of the egg, statistical results (*p* < 0.05) were obtained in terms of yolk weight, with the treatment with 0.4% showing better results compared to the 0% and 0.2% PCCP treatments, but not with the 0.6% PCCP treatment. The other variables evaluated did not show statistically variable results (*p* > 0.05); however, numerically, the treatment with 0.4% PCCP obtained a higher albumen weight compared to the other treatments, and the treatments with 0.2% and 0.4% obtained higher values in terms of Haugh units compared to the eggs from the control treatment.

The results of this study indicate that anthocyanins, which have antioxidant and antimicrobial characteristics [43,44], allow greater assimilation of nutrients and energy and consequently, better efficiency and availability of nutrients for increasing weight and egg mass [35].

Regarding the evaluation of the internal quality of the egg, the albumin index and Haugh units are essential parameters. The quality of the albumen depends on the *β*-ovomucin polymers amount secreted by the magnum, in addition to the ovomucin-lysozyme complex, and their structures are some of the main determinants of the gelatinous property of the albumen and its height [45,46]. Previous studies have shown that the antioxidant properties of polyphenols (in this case, anthocyanins) added to the diet of the hens can protect the structure of these albumen components [47]. Albumen is an important water reserve of the egg, which also makes it an equal contributor to proteins [48]. Given that anthocyanin pigment is classified into water-soluble flavonoids [49], the significant effect shown is probably due to the incorporation of anthocyanin pigment into the egg albumen.

**Table 5.** Effects of the addition of different percentages of purple corn cob powder (PCCP) on the external and internal quality in eggs of laying hens.

Egg quality	Treatments				SEM	*p*-value
	0%	0.2%	0.4%	0.6%		
External quality						
Egg weight (gm)	63.22^b^	63.88^ab^	68.54^a^	66.62^ab^	2.487	0.0362
Egg mass (gm)	50.21^b^	50.42^b^	62.43^a^	56.51^ab^	3.230	0.0005
Internal quality						
Shell weight (gm)	6.28	6.45	6.63	6.65	0.469	0.6727
Albumen weight (gm)	40.38	40.35	42.72	41.32	2.338	0.4646
Yolk weight (gm)	16.56^b^	17.08^b^	19.19^a^	18.66^a^	0.712	0.0006
Morphological index	72.07	75.96	77.17	75.85	5.413	0.5882
Shell index	8.15	8.73	8.72	8.69	0.507	0.3416
Yolk index	42.25^b^	44.75^ab^	46.25^a^	45^ab^	0.019	0.0286
Shell thickness (mm)	0.41	0.44	0.42	0.43	0.029	0.6513
Yolk color (Roche)	8.75	9.19	9.91	9.69	0.676	0.1227
Specific gravity	1.09	1.09	1.09	1.09	0.002	0.5933
Haugh units	86.47	94.38	94.22	93.91	5.959	0.2256

*N* = 56 eggs per treatment per week; Treatments = Treatment 1-0%PCCP, Treatment 2-0.2% PCCP, Treatment 3-0.4% PCCP, and Treatment 4-0.6% PCCP; Roche = Roche colorimetric fan, ^ab^ = Different letters in each average indicate significant differences between treatments with the Tukey test (*p* < 0.05).

Eggs contain different antioxidant agents such as lysozyme, ovalbumin, ovotransferrin peptides, egg white amino acids, carotenoids, phosvitin, vitamin E, and free aromatic amino acids from egg yolk [50]. On the other hand, egg white contains ovoinhibitor, a serine protease inhibitor that can reduce enzymatic digestion by trypsin and chymotrypsin.

The inclusion of PCCP has shown significant effects on the external and internal quality of the egg compared to a control group. Similar results have been found with the use of various sources of anthocyanins in hens, thus supporting the significant increase in weight and egg mass (*p* < 0.05) with the inclusion of 0.2% pomegranate peel, 5%, 10%, and 15% pomegranate seed pulp, 2% açaí flour, 1, 5, 10, and 20 gm/kg of Goji berry leaves, 4% and 6% grape pomace, and 0.4% and 4% hibiscus calyx powder in the feed [14,35–38]. However, Duru [37] and Musa-Azara et al. [40] found no significant differences (*p* > 0.05) in egg weight by the addition of goji berry leaves to the diet and hibiscus calyx extract in the drinking water of laying hens. Additionally, they found no differences in the weight of albumen, yolk, or eggshell.

Regarding the albumen index, Duru [37] reported a significant increase (*p *< 0.05) in this index with the addition of 1 and 5 gm/kg Goji berry in the diet. This could directly affect the Haugh units, which were significantly increased compared to a control diet in the studies carried out by Kaya et al. [34], with the inclusion of grape seed extract. Conversely, Saki et al. [35], Kara et al. [14] and Sukkhavanit et al. [36] reported that by adding pomegranate seed pulp at levels of 5%, 10%, and 15%, grape pomace at 4% and 6%, and extract and hibiscus calyx flour, respectively, to the diet of laying hens, the Haugh units were not affected.

The use of 2%, 4%, and 6% hibiscus flower powder [13] and 5% dry grape peel [51], respectively, did not alter the yolk color according to the Royal Yolk Fan Color score, with all treatments having an average score of nine. On the other hand, studies conducted in quails presented results such as those obtained by adding PCCP to the diet, as indicated by Boontiam et al. [39], who observed improvements in Haugh units with the addition of mangosteen pericarp extract.

### External and internal egg quality during storage

Table 6 presents the results regarding egg weight loss during storage for hens supplemented with PCCP. Regarding weight loss on days 14, 21, 28, and total weight loss, statistically significant differences (*p* < 0.05) were observed, with superior results for diets supplemented with PCCP compared to the control diet on days 14 and 21. However, on day 28, the treatment with 0.6% PCCP achieved better results compared to the control treatments and those including 0.2% PCCP. Regarding total weight loss, treatments with 0.4% and 0.6% PCCP obtained better results compared to the other treatments.

**Table 6.** Effects of the addition of different percentages of purple corn cob powder (PCCP) on egg weight loss during storage at room temperature (21°C).

Storage days	Treatments				SEM	*p*-value
	0%	0.2%	0.4%	0.6%		
1	65.37^b^	66.37^ab^	66.43^ab^	67.46^a^	0.743	0.0534
7	64.93^b^	65.68^ab^	66.11^ab^	66.87^a^	0.711	0.0547
14	64.01^b^	65.19^ab^	65.66^a^	66.06^a^	0.611	0.0165
21	62.78^b^	65.01^a^	65.01^a^	65.42^a^	0.823	0.0165
28	61.84^c^	62.96^bc^	64.33^ab^	65.01^a^	0.621	0.0011
Total loss (1-28 days)	3.53^a^	3.41^ab^	2.10^c^	2.45^bc^	0.383	0.0041
Total loss 1-28 days (%)	5.39^a^	5.15^a^	3.15^b^	3.64^b^	0.562	0.0028

Treatments = Treatment 1-0% PCCP, Treatment 2-0.2% PCCP, Treatment 3-0.4% PCCP, and Treatment 4-0.6% PCCP; ^abc ^= different letters in each row indicate significant differences between treatments for the Tukey test (*p* < 0.05).

Table 7 presents the results regarding the weight loss of yolk, albumen, and eggshell on days 1 and 28 of storage, as well as the difference in Haugh units on days 1 and 28 of storage. No statistically significant results were found (*p *> 0.05) with respect to yolk weight loss.

It is important to mention the weight loss of eggs in storage, as this can affect their internal quality. There is limited information regarding this evaluation; however, studies with the most notable results indicate that the inclusion of 1% açaí flour induces a lower reduction in weight in eggs stored at room temperature for 28 days [5]. Similarly, Ryu and No [52] reported that covering eggs stored at room temperature with grape seed oil lessens weight loss (*p *< 0.05), being 12.5 times less compared to the control group (0.58% vs. 7.26%, respectively).

Regarding the weight loss of the albumen, different results (*p* < 0.05) were found on day 28 of storage, with the treatment of 0.4% PCCP showing the least weight loss compared to the control treatment. Numerically, in terms of total loss (%), treatments with 0.4% and 0.6% PCCP obtained better results compared to the other treatments. No differences were observed in the weight loss of the shell among the different treatments (*p* > 0.05). Finally, regarding Haugh units, there was a statistically significant difference (*p *< 0.05) on day 28 of storage, with PCCP treatments of 0.4% compared with 0 and 0.2% PCCP. However, there were no statistical differences (*p* > 0.05) with respect to the diet with 0.6% PCCP.

**Table 7.** Results of yolk, albumen, eggshell weight loss, and Haugh units on days 1 and 28 of storage in eggs from laying hens supplemented with different percentages of purple corn cob powder (PCCP).

Weight loss/day		Treatments				SEM	*p*-value
		0%	0.2%	0.4%	0.6%		
Yolk	1	17.18	17.87	18.26	17.96	0.707	0.3506
	28	16.46	17.16	17.71	17.43	0.729	0.2616
	Total (%)	4.15	4.02	3.00	2.96	0.529	0.0427
Albumen	1	41.30	42.00	41.30	42.60	0.707	0.1388
	28	38.91^a^	39.50^ab^	39.51^ab^	40.81^b^	0.604	0.0265
	Total (%)	5.14	4.81	3.23	3.21	0.990	0.0839
Shell	1	7.33	7.00	7.30	7.30	0.500	0.8018
	28	6.46	6.30	6.76	6.76	0.397	0.4329
	Total (%)	0.70	0.70	0.56	0.56	0.370	0.9395
Haugh units	1	90.00	90.30	90.30	90.30	1.000	0.9672
	28	77.66^b^	78.30^b^	81.60^a^	82.00^a^	1.732	0.0307

Treatments = Treatment 1-0% PCCP, Treatment 2-0.2% PCCP, Treatment 3-0.4% PCCP, and Treatment 4-0.6% PCCP; ^ab^ = Different letters in each row indicate significant differences between treatments with the Tukey test (*p* < 0.05).

These results are supported by those of Fortuoso et al. [5], who found no differences in Haugh units among groups of fresh or stored eggs (*p* > 0.05) with different percentages of açaí flour supplementation. In general, Haugh units decrease with longer storage periods; however, this reduction progresses more slowly in eggs covered with oil compared to uncovered eggs, showing significantly higher Haugh units and preservation of albumen quality. A similar situation was observed with the inclusion of PCCP in the present study, with significant differences being found at 28 days of egg storage at room temperature (21°C), especially within the group receiving 0.6% PCCP.

The main changes in storage were observed in relation to the weight loss of albumen. Nonetheless, the decrease in Haugh units was not high compared to day 1, especially in the treatments with 0.4 and 0.6% PCCP.

### Total solids and bromatological analysis

Table 8 shows the nutritional analysis of the egg yolk, with different effects (*p* > 0.05) according to the protein content and the ether extract. Thus, the protein content was higher in the eggs from hens fed with 0.4% PCCP, while the ether extract was higher in the control treatment and birds fed with 0.2% PCCP.

***TBARS levels in egg yolk***

Table 9 presents the results of TBARS levels in egg yolk on both days 1 and 28 of storage. There were no statistically significant differences on day 1 (*p* > 0.05), while egg yolks from the 0.4% and 0.6% PCCP treatments showed lower lipid peroxidation on day 28.

Lipid oxidation is an inevitable natural phenomenon that occurs in the egg yolk, directly affecting its commercial value during storage and its nutritional value, color, flavor, and texture [53]. The oxidation of polyunsaturated fatty acids (PUFAs), which are more susceptible to this due to having several double bonds [54], begins with the creation of a free radical chain reaction by the exposure of lipids to light, heat, or ionizing radiation. Antioxidants, such as anthocyanins, can stop this chain by reacting with free radicals and as metal chelators, hence the need to protect PUFAs using antioxidants [55,56].

Lipid peroxidation in the yolk leads to the production of MDA, which is harmful to human and animal health [57,58]. Anthocyanins can penetrate cell membranes to participate and stimulate antioxidant enzymes (superoxide dismutase, glutathione peroxidase, and catalase) [30,44], which can directly contribute to reducing lipid peroxidation of the yolk, as these antioxidants can accumulate in the egg yolk. Furthermore, the anthocyanins can decrease the frequency of primary oxidation product concentrations such as diene and triene conjugates, thereby reducing MDA values [12].

The egg albumen and egg yolk contain about 7.04 and 62.416 ppm of iron [59], and the range of iron for metal-catalyzed oxidation is from 1 ppb to 500 ppm in the egg as reported by Labuza et al. [60], explaining the susceptibility of the egg yolk to lipid oxidation. The egg yolk contains phosvitin, an iron chelator that can inhibit Fe^2+^-catalyzed phospholipid oxidation and can act as a natural antioxidant, aiding in the preservation of the characteristics of the yolk [61].

**Table 8.** Total solids and bromatological analysis in egg yolks from hens supplemented with different percentages of purple corn cob powder (PCCP).

Indexes	Treatments				SEM	*p*-value
	0%	0.2%	0.4%	0.6%		
Fresh egg moisture (%)	49.87	49.64	48.02	48.76	0.071	0.0672
Fresh total solids (%)	50.13	50.36	51.98	51.24	1.340	0.1352
Freeze-dried moisture (%)	3.95	3.80	3.09	3.84	0.320	0.0973
Freeze-dried total solids (%)	96.05	96.20	96.91	96.16	0.298	0.2430
Protein (%)	30.16^b^	30.52^b^	31.97^a^	30.96^b^	0.141	0.0041
Ether extract (%)	54.36^a^	54.13^a^	53.46^b^	53.72^b^	0.122	0.0356
Ashes (%)	3.32	3.47	3.59	3.52	0.093	0.2520
Nitrogen-free extract (NFE) (%)	8.21	8.08	7.89	7.96	0.883	0.0582

Treatments = Treatment 1-0% PCCP, Treatment 2-0.2% PCCP, Treatment 3-0.4% PCCP, and Treatment 4-0.6% PCCP; ^ab^ = Different letters in each average indicate significant differences between treatments with the Tukey test (*p* < 0.05).

**Table 9.** TBARS Test in egg yolks from hens supplemented with purple corn cob powder (PCCP), corresponding to the final day of the experiment and in storage for 28 days.

Oxidation	Treatments				SEM	*p*-value
	0%	0.2%	0.4%	0.6%		
Day 1 (μmoles/mg)	0.1423	0.1443	0.1431	0.1440	0.006	0.9210
Day 28 (μmoles/mg)	0.3858^a^	0.3778^a^	0.3293^b^	0.3290^b^	0.013	0.0001

Oxidation: obtained by the TBARS methodology; Treatments = Treatment 1-0% PCCP, Treatment 2-0.2% PCCP, Treatment 3-0.4% PCCP, and Treatment 4-0.6% PCCP; ^ab^ = Different letters in each average indicate significant differences between treatments with the Tukey test (*p* < 0.05).

This fact is important, as peroxidation leads to a rancid taste and, consequently, a decrease in the sensory and nutritional quality of the egg. Moreover, the quality of the eggs was preserved, even when stored for 28 days in refrigeration (6°C), increasing their shelf life. This demonstrates that the inclusion of anthocyanins improves the quality and lengthens the durability of the eggs and can also provide benefits for the health of the hens. It should be noted that results regarding TBARS levels by the inclusion of anthocyanins may vary depending on the source of the polyphenols used and where they were harvested, as well as variations in their processing, the percentage of supplementation, and the type of diet provided to the animals [14].

Lipid peroxidation levels are important for assessing the quality and nutritional value of egg yolks. In the present study, the inclusion of PCCP at 0.4 and 0.6% in the diet of laying hens showed lower TBARS levels (*p* < 0.05) compared to the control group at 28 days of storage at 6°C. These findings are supported by those of Sukkhavanit et al. [36], who reported that with the inclusion of 2% hibiscus calyx extract and 2% hibiscus calyx flour, TBARS values significantly decreased compared to the control group in eggs stored in refrigeration. They also evaluated TBARS values in eggs stored outdoors, presenting higher values than the refrigerated group at 20 days of storage.

### Fatty acid profile in egg yolk

Table 10 presents the results of the number of fatty acids present in the egg yolks of hens receiving different treatments with varying percentages of PCCP supplementation. Regarding egg weight, different results were obtained in the percentage of total lipids, myristic acid, palmitic acid, stearic acid, and palmitoleic acid (*p* < 0.05), with better outcomes for treatments of 0.4% and 0.6% PCCP compared to the two other groups. Saturated and unsaturated fatty acids (*p* < 0.05) showed better outcomes with the 0.4% PCCP treatment compared to the other treatments.

**Table 10.** Fatty acid profile in freeze-dried egg yolks from laying hens supplemented with different percentages of purple corn cob powder (PCCP).

Fatty acids	Treatments				SEM	*p*-value
	0%	0.2%	0.4%	0.6%		
Egg weight (gm)	16.35^b^	16.39^b^	17.66^a^	17.12^a^	0.258	0.0006
Total lipids (%)	31.95^b^	31.99^b^	33.07^a^	32.32^ab^	0.307	0.0073
Saturated fatty acids						
Myristic (C14:0)	0.223^a^	0.163^ab^	0.126^c^	0.146^bc^	0.012	0.0001
Palmitic (C16:0)	24.92^a^	24.53^ab^	24.24^b^	24.23^b^	0.213	0.0126
Stearic (C18:0)	8.61^a^	8.44^ab^	8.01^b^	8.16^ab^	0.179	0.0132
Unsaturated fatty acids						
Palmitoleic (C16:1)	1.76^b^	1.76^b^	1.87^a^	1.873^a^	0.039	0.0078
Oleic (C18:1)	41.26^c^	41.66^bc^	42.93^a^	42.16^b^	0.294	0.0007
Linoleic (C18:2)	16.29^c^	16.35^c^	18.14^a^	17.47^b^	0.179	0.0001
Linolenic (C18:3)	0.099	0.098	0.1173	0.123	0.010	0.0331

Treatments = Treatment 1-0% PCCP, Treatment 2-0.2% PCCP, Treatment 3-0.4% PCCP, and Treatment 4-0.6% PCCP; ^abc^ = Different letters in each average indicate significant differences between treatments with the Tukey test (*p* < 0.05).

This desirable effect observed in terms of the quantity of both saturated and unsaturated fatty acids may be related to the reduction of lipid peroxidation and the increase in antioxidant levels in the eggs (Table 10), as the lipoperoxidation process affects the fatty acid profile. The low content of myristic (C14:0), palmitic (C16:0), and stearic (C18:0) acids is associated with cardiovascular diseases in humans due to the increase in low-density lipoproteins [62].

The effects of antioxidants on health and performance, along with their impact on the fatty acid profile and overall antioxidant capacity of eggs, are significant [63]. It was observed that the inclusion of 0.4% PCCP in the diet of laying hens presents better values regarding an increase of unsaturated fatty acids. The addition of purple corn anthocyanins to the diet of broiler chickens led to a statistically significant reduction (*p* < 0.05) in some concentrations of saturated fatty acids, such as myristic acid (C14:0), compared to the control group [25].

However, they found no differences (*p* > 0.05) in muscle levels of oleic acid (C18:1), linoleic acid (C18:2), or linolenic acid (C18:3). Similar results were obtained in the studies by Fortuoso et al. [5] and Kara et al. [14], in which the inclusion of 1% and 2% açaí flour and 4% grape pomace, respectively, in the diet of laying hens led to an alteration in fatty acid values, reducing stearic acid (C18:0) and increasing linoleic acid (C18:2) and linolenic acid (C18:3).

## Conclusion

The inclusion of PCCP in the diet of laying hens positively affects the productive indices of external and internal quality of the eggs, finding statistically significant results in the inclusion of 0.4% and 0.6% of PCCP. An increase in egg production and an improvement in the FCR were demonstrated. Additionally, an increase in egg weight and mass, as well as the weight of albumen and yolk, enhancing Haugh units at 28 days storage, was achieved. In addition, an increase in unsaturated fatty acids in the yolk was observed. Finally, a decrease in weight loss during storage for 28 days at room temperature (21°C) and lower levels of lipid peroxidation in the yolk were also evidenced. Therefore, the addition of PCCP to the diet of laying hens could contribute to the production of eggs with better nutritional value and with a slower loss of characteristics when stored, being beneficial for the consumer. Nonetheless, further research on the mechanisms of action of anthocyanins regarding the changes generated is necessary.

## List of abbreviations

DF, dilution factor; AOAC, Association of Official Analytical Chemists; FCR, feed conversion ratio; MDA, malondialdehyde; MW, molecular weight; PCCP, purple corn cob powder; pH, hydrogen potential; PUFA, polyunsaturated fatty acids; RH, relative humidity; TBARS, thiobarbituric acid reactive substances.
